# Fourteen-day administration of corticosterone may induce detrusor overactivity symptoms

**DOI:** 10.1007/s00192-016-3027-3

**Published:** 2016-05-03

**Authors:** Andrzej Wróbel, Anna Serefko, Ewa Poleszak, Tomasz Rechberger

**Affiliations:** 1 Second Department of Gynecology, Medical University of Lublin, Jaczewskiego 8, 20-090 Lublin, Poland; 2 Chair and Department of Applied Pharmacy, Medical University of Lublin, Chodźki 1, 20-093 Lublin, Poland

**Keywords:** Corticosterone, CRF_1_ antagonist, Depression, Overactive bladder, Rats

## Abstract

**Introduction and hypothesis:**

Epidemiological studies demonstrated that patients suffering from overactive bladder often present with different mental problems, amongst which depression is the most frequently observed. The main goal of our study was to check if the repeated administration of corticosterone (CORT) is able to evoke the depressive-like behaviour and detrusor overactivity (DO) symptoms in rats. Moreover, we investigated whether the acute administration of common antidepressants (imipramine, 30 mg/kg, and fluoxetine, 15 mg/kg), antimuscarinic (oxybutynin chloride, 0.5 mg/kg) or CRF_1_ (SN 003, 1 mg/kg) antagonists has an impact on the cystometric parameters, behaviour in the Porsolt test, and overall locomotor activity of animals exposed to CORT.

**Methods:**

The experiments were carried out on female Wistar rats. All applied surgical and histopathology procedures, cystometric investigations, locomotor activity and forced swim measurements have been fully described in the published literature.

**Results:**

Fourteen-day administration of CORT may induce both depressive and DO symptoms in rats, which are reversed by the inhibition of CRF_1_ receptors.

**Conclusions:**

It seems that the CRF_1_ receptor could be an interesting target for overactive bladder pharmacotherapy, particularly in patients with co-existing depression.

## Introduction

Overactive bladder (OAB) is a symptom-based diagnosis defined by the International Continence Society as urinary urgency with (OAB wet) or without (OAB dry) urgency incontinence, usually with frequency and nocturia in the absence of local pathological lesions that could cause the aforementioned symptoms [1]. The incidence of OAB amounts to 16.9 % and increases with age, reaching 30.9 % in the group of people aged above 65 [2]. Therefore, it is considered a social disease with an incidence almost equal to heart diseases, hypertension, and bronchial asthma [2]. Although OAB is not a life-threatening disease, it is known to have a significant impact on the quality of life, as it causes sleeping disorders and depression. Patients suffering from OAB often present with different mental problems, amongst which depression is the most frequently observed. The link between depression and OAB was described for the first time in 1964. To date, a non-formal term, “uropsychiatry”, which emphasises a close association between these two diseases, has been used in the medical literature [3].

Epidemiological studies demonstrated that depression is an independent risk factor for OAB. It was observed that in a group of patients aged 65 and older, 42 % of the patients with depression already diagnosed presented with OAB symptoms, in comparison with 13 % of patients who exhibited stress urinary incontinence (SUI) symptoms. Sixty percent of patients with urge incontinence symptoms had Beck’s Depression Inventory scores >12 [4]. Moreover, other authors indicated a positive correlation between the onset of depressive symptoms and the presence of OAB [3]. Based on the results of clinical trials, patients with OAB have worse scores on the Centre of Epidemiologic Studies Depression (CES-D) scale in comparison with people with normal urination [2]. A statistically significant increase in the frequency of depression in patients with OAB (wet or dry) was observed in the Epidemiology Urinary Incontinence and Comorbidities (EPIC) study. Similarly, the results of the Epidemiology of Lower Urinary Tract Symptoms Study (EpiLUTS) demonstrated that patients with OAB are 27 % more likely to suffer from this affective disorder [5].

Despite a close and clinically confirmed association between depression and OAB, whether this affective disorder is an OAB-inducing factor or whether OAB is a specific manifestation of psychosomatic disorders has still not been resolved. Co-existence of some neurochemical dysfunctions that underlie the aetiopathogenesis of both diseases cannot be ruled out. It was proved that the dysregulation of serotonergic or adrenergic neurotransmission in addition to abnormalities in the function of corticotropin-releasing factor (CRF) may play a significant role in the pathophysiology of both depression and OAB [6].

The aim of our study was to check if the 14-day administration of corticosterone (CORT), which induced depressive-like behaviour in pre-clinical studies, is also able to evoke detrusor overactivity (DO) symptoms [7–9]. At the same time, we wanted to investigate whether the administration of antidepressants (imipramine, fluoxetine) and antimuscarinic or CRF_1_ antagonists (oxybutynin chloride or SN 003 respectively) has an impact on the cystometric parameters, the locomotor activity of animals, in addition to their behaviour in the forced swim test (FST).

## Materials and methods

All procedures were conducted according to NIH Animal Care and Use Committee guidelines, and were approved by the Ethics Committee of the Medical University of Lublin.

### Animals

The study was conducted on female Wistar rats (initially weighing 200–225 g). A natural light/dark cycle, temperature of 22 °C and humidity of 60 % were maintained. Food and water were provided ad libitum. All experimental procedures were carried out between 8 a.m. and 1 p.m. Rats were experimentally naive and tested only once. A total of 90 female Wistar rats were used and randomly assigned to one of the six following treatment groups of 15 rats each:

1. Control group receiving physiological saline for 14 days (CON)
2. Corticosterone 20 mg/kg/day for 14 days (CORT)
3. CORT plus imipramine 30 mg/kg (CORT + IMI)
4. CORT plus fluoxetine 15 mg/kg (CORT + FLX)
5. CORT plus CRF_1_ antagonist 1 mg/kg (CORT + SN003)
6. CORT plus oxybutynin chloride 0.5 mg/kg (CORT + OXY)

Each rat was placed in a metabolic cage (3700 M071; Tecniplast, West Chester, PA, USA) with free access to food and water.

### Drugs

The following drugs were used: oxybutynin chloride (OXY; Sigma-Aldrich), imipramine (IMI; Polpharma), fluoxetine (FLX; Eli Lilly), corticosterone (CORT; Tocris), and SN 003 (N-(4-Methoxy-2-methylphenyl)-1-[1-(methoxymethyl)propyl]-6-methyl-1H-1,2,3-triazolo[4,5-c]pyridin-4-amine; Tocris). The doses of the agents administered were taken from the literature and had been confirmed/adjusted in our laboratory in preliminary experiments. OXY (Sigma-Aldrich) was dissolved in a volume of 1 ml/kg with 0.5 % hydroxypropyl methyl cellulose (HPMC; Sigma-Aldrich) solution containing 0.1 % Tween 80 (Sigma-Aldrich) and administered intravenously as a single dose of 0.5 mg/kg via a polyethylene catheter inserted into the right femoral vein. IMI (30 mg/kg) and FLX (15 mg/kg) were given intraperitoneally (i.p.) 60 min before the tests. SN 003 was dissolved in DMSO in a volume of 1 ml/kg, and was given intravenously at a dose of 1 mg/kg. CORT (20 mg/kg/day) was given subcutaneously (s.c.) for 14 days.

### Surgical procedures

All the surgical procedures were performed as described previously [10], under anaesthesia with i.p. injection of 75 mg/kg of ketamine hydrochloride (Ketanest; Pfizer) and 15 mg/kg of xylazine (Sedazin; Biowet). Rats were placed supine on a warming mattress (37 °C). Lack of spontaneous movement and lack of withdrawal response to a noxious toe pinch served as markers for adequate depth of anaesthesia.

The shaved and cleaned abdominal wall was opened through an approximately 10-mm vertical midline incision. The bladder was gently freed from the adherent tissues. A double lumen polyethylene catheter (inside diameter, i.d., 0.28 and outside diameter, o.d., 0.61 mm; BD, Franklin Lakes, NJ, USA), filled with physiological saline with a cuff at the end was inserted through a small incision into the apex of the bladder dome and fixed with a 6-0 Vicryl suture. In the same session the right femoral vein was catheterised via an inguinal approach. A polyethylene catheter (i.d. 0.28 and o.d. 0.61 mm; BD) filled with 40 IU/ml of heparinised physiological saline for the infusion of test compounds or physiological saline into the bloodstream was inserted into the vessel and advanced proximally until the tip of the catheter reached the abdominal aortic bifurcation. The catheters were tunnelled subcutaneously and exteriorised in the retroscapular area, where they were connected with a plastic adapter, to avoid the risk of removal by the animal. The chronically implanted intravenous catheter ensured stress-free conditions during the experiment. Finally, Healon (Pharmacia A.B.) at a dose of 0.85 ml was applied around the urinary bladder to avoid adhesions. The abdomen was closed in multiple layers. Anatomical layers were closed using 4/0 catgut sutures. The free ends of the catheters were sealed with silk ligatures. The animals were injected subcutaneously with 100 mg of cefazolin sodium hydrate (Biofazolin; Sandoz) to prevent urinary tract infection.

### Conscious cystometry

Cystometric investigations were performed in conscious unrestrained rats 3 days after surgical procedures. The bladder catheter was connected via a three-way stopcock to a pressure transducer (FT03; Grass Instruments) situated at the level of the bladder and to a microinjection pump (CMA 100; Microject, Solna, Sweden) for recording intravesical pressure and for infusing physiological saline into the bladder. Conscious cystometry was performed by slowly filling the bladder with physiological saline (at a constant rate of 0.05 ml/min, i.e. 3 ml/h) at room temperature (22 °C) to elicit repetitive voiding. The infusion rate was based on pilot studies in which the rate of 0.05 to 0.1 ml/min was associated with bladder cystometry profiles similar to those in the intact lower urinary tract in rats [11]. Higher infusion rates usually resulted in an increase in bladder capacity or a reflex contraction of the bladder detrusor muscle. The analogue signal obtained from the pressure transducer was amplified and digitised using the Polyview system (Grass Instruments). Micturition volumes were measured by means of a fluid collector attached to a force displacement transducer (FT03C; Grass Instruments). Both transducers were connected to a polygraph (7 DAG; Grass Instruments). Cystometry profiles and micturition volumes were recorded continuously on a Grass polygraph (Model 7E; Grass Instruments) and were determined graphically. The data were analysed using a sampling rate of 10 samples/s. The measurements in each animal represent the average of five bladder micturition cycles after obtaining repetitive voiding. All procedures were performed by a colleague who was blinded to the treatments. At the end of the experiments, rats were euthanised with CO_2_ gas.

The following cystometric parameters were recorded:

- Basal pressure (BP, cm H_2_O): the lowest bladder pressure during the filling phase
- Intercontraction interval (ICI, s): the interval between the maximum detrusor pressure (Pdet) during micturition and the next maximum Pdet during micturition
- Bladder compliance (BC, ml/cm H_2_O): calculated as the bladder capacity divided by the difference in the pressure threshold and baseline pressure using the formula (VV + PVR)/(TP − BP)
- Detrusor overactivity index (DOI, cm H_2_O/ml): depicted as the quotient of the sum of amplitudes of all detrusor contractions during the filling phase and functional bladder capacity [10]
- Non-voiding contractions (NVCs): frequency (FNVC, times/filling phase) and amplitude (ANVC, cm H_2_O): an increase in bladder pressure without the release of fluid from the urethra. Non-voiding contractions (vesical pressure increases before each micturition without expulsion of the fluid) higher than 2 cm H_2_O were used as a surrogate for detrusor overactivity [12]. A voiding contraction was identified as a large increase in bladder pressure accompanied by the release of fluid from the urethra
- Volume threshold to elicit NVC (VTNVC, %): percentage of total bladder filling volume, which is the preclinical equivalent to the volume at first involuntary detrusor contraction measured during urodynamic investigations in humans [13]
- Voided volume (VV, ml): volume of expelled urine
- Post-void residual (PVR, ml): bladder capacity minus voided volume/fluid remaining in the bladder at the end of micturition

### Locomotor activity

The locomotor activity of rats was assessed using a a Digiscan apparatus: an Optical Animal Activity Monitoring System (Omnitech Electronics, Columbus, OH, USA), which monitored animal locomotor activity via invisible infrared light beams. The interruption of the beam by a tested rat was recorded as an activity score. Horizontal activity, defined as the total number of beam interruptions that occurred in the horizontal sensor during 1 h of measurement, was assessed. Before behavioural analysis, animals were placed into activity chambers for a 15-min habituation period.

### Forced swim test

The FST was carried out according to the method described in detail by Porsolt et al. [14]. Rats were placed individually into glass cylinders (height 65 cm, diameter 25 cm) containing 48 cm of water (maintained at 23–25 °C) for 15 min (pre-test) before being returned to their home cages. After 24 h, animals were retested for 5 min under identical swim conditions.

### Histopathology

The bladders of the animals tested were removed, fixed in 10 % neutral buffered formalin for 24 h and processed for paraffin block. The bladder wall sagittal sections (5 μm in thickness) from the dome to the trigone were cut and stained with haematoxylin and eosin.

### Study design

Rats from groups 2–6 underwent administration with CORT s.c. at a dosage of 20 mg/kg/day in a volume of 1 ml/kg for 14 days. The animals from group 1 underwent s.c. administration of an equivalent volume of physiological saline. On the 14th day, in all animals from groups 1–6, the surgical procedures described above were performed. After 3 days, the following studies were carried out: cystometry, the Porsolt test and locomotor activity measurement. Immediately after the behavioural test, animals’ blood was collected by cardiac puncture. The CRF level was assessed using a commercially available enzyme immunoassay (LBS), according to the manufacturer’s instructions.

### Statistical analysis

The data obtained were assessed by *t* test or one-way analysis of variance (ANOVA) followed by Tukey’s post hoc test, depending on the experimental design. All results are presented as the means ± standard error of the mean (SEM).* p* < 0.05 was considered to indicate a statistically significant difference.

## Results

### Cystometric study

Fourteen-day administration of CORT at a dose of 20 mg/kg/day induced changes in the cystometric parameters specific to DO. An increase in the following parameters was recorded: BP (F(5.84) = 12.29;* p* < 0.001), DOI (F(5,84) = 19.30;* p* < 0.001), ANVC (F(5,84) = 15.67;* p* < 0.001), and FNVC (F(5,84) = 20.86;* p* < 0.001), whereas a decrease in VV (F(5,84) = 11.20;* p* < 0.01), ICI (F(5,84) = 11.79;* p* < 0.01), BC (F(5,84) = 12.84;* p* < 0.001), and VTNVC (F(5,84) = 5.845;* p* < 0.001) was observed. PVR did not change significantly. Administration of both IMI and FLX did not evoke a considerable alteration of the cystometric parameters in animals with CORT-induced DO. On the other hand, SN 003 reversed DO-specific changes in the cystometric parameters in animals, resulting in an increase in VV (F(5,84) = 11.20;* p *< 0.01), ICI (F(5,84) = 11.79;* p* < 0.05), BC (F(5,84) = 12.84; p < 0.05), and VTNVC (F(5,84) = 5.845;* p* < 0.05), in addition to inducing a decrease in BP (F(5,84) = 12.29;* p* < 0.001), DOI (F(5,84) = 19.30;* p* < 0.001), ANVC (F(5,84) = 15.67;* p* < 0.01), and FNVC (F(5,84) = 20.86;* p* < 0.001). SN 003 did not have a significant impact on PVR. Administration of OXY led to similar effects to those observed for SN 003, except for PVR value. After injection of the muscarinic receptor antagonist a significant increase in PVR was noted (F(5,84) = 5.535;* p* < 0.05). The summarised results are presented in Table 1.

**Table 1:** The influence of the repeated administration of corticosterone (CORT) on the cystometric parameters in conscious rats

	BP cm H_2_O	VV ml	PVR ml	ICI s	BC ml/ cm H_2_O	DOI cm H_2_O/ml	ANVC cm H_2_O	FNVC times/ filling phase	VTNVC %
CON	2.947 ± 0.160	0.842 ± 0.039	0.076 ± 0.004	902.3 ± 18.71	0.189 ± 0.007	114.7 ± 4.732	2.407 ± 0.081	0.861 ± 0.122	47.93 ± 1.909
CORT	5.033 ± 0.359***	0.610 ± 0.052**	0.065 ± 0.004	716.0 ± 38.60**	0.136 ± 0.009***	312.9 ± 29.59***	5.940 ± 0.334***	7.107 ± 0.837***	35.07 ± 1.926***
CORT + IMI	4.480 ± 0.240	0.554 ± 0.033	0.066 ± 0.005	652.3 ± 37.23	0.130 ± 0.008	319.5 ± 25.39	5.207 ± 0.389	5.793 ± 0.564	36.87 ± 1.393
CORT + FLX	4.560 ± 0.244	0.545 ± 0.036	0.053 ± 0.003	695.1 ± 41.04	0.114 ± 0.008	274.6 ± 25.25	5.647 ± 0.500	5.740 ± 0.513	40.67 ± 2.643
CORT + SN 003	3.353 ± 0.222^^^	0.822 ± 0.045^^	0.071 ± 0.006	869.9 ± 34.61^	0.177 ± 0.008^	188.3 ± 15.95^^^	4.253 ± 0.349^^	3.667 ± 0.341^^^	44.13 ± 2.671^
CORT + OXY	3.240 ± 0.197^^^	0.814 ± 0.044^	0.087 ± 0.004^	911.9 ± 27.27^^	0.176 ± 0.008^	141.6 ± 5.198^^^	3.653 ± 0.223^^^	3.253 ± 0.219^^^	46.20 ± 1.993^^

All results are presented as the means ± SEM (*n* = 15 rats per group)

The data obtained were assessed by one-way analysis of variance (ANOVA) followed by Tukey’s post hoc test

**p < 0.01 versus CON, ***p < 0.001 versus CON, ^*p *< 0.05 versus CORT, ^^*p *< 0.01 versus CORT, ^^^*p* < 0.001 versus CORT

### Locomotor activity

**Fig. 1:**
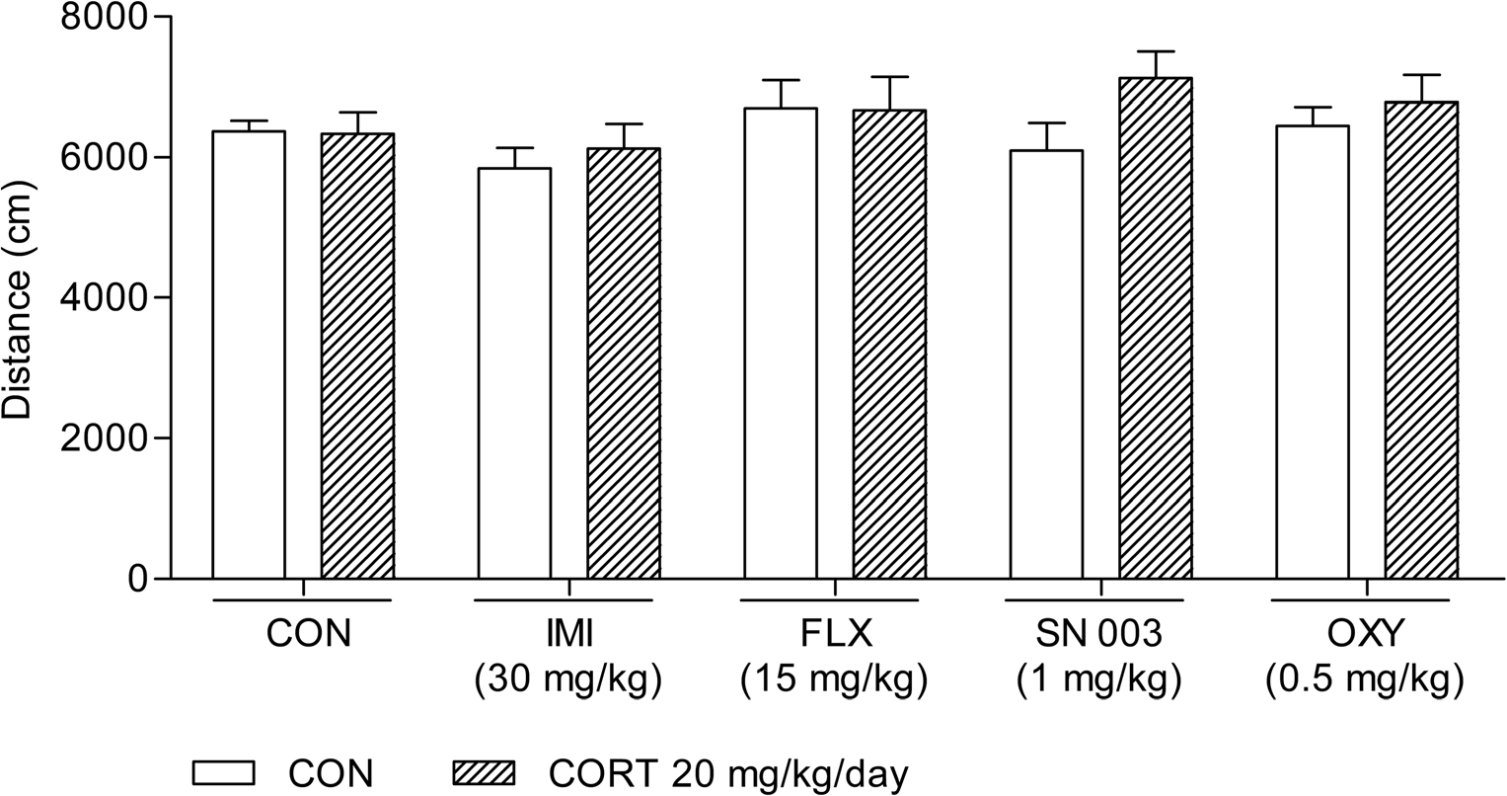
Influence of the acute administration of imipramine (*IMI*, 30 mg/kg), fluoxetine (*FLX*, 15 mg/kg),* SN 003* (1 mg/kg), and oxybutynin chloride (*OXY*, 0.5 mg/kg) on the locomotor activity of rats subjected to 14-day corticosterone treatment (*CORT*, 20 mg/kg/day). The values represent the mean + SEM (*n* = 15 mice per group).* CON* control

None of the tested agents given alone or in the combinations used influenced the locomotor activity of animals compared with the control group (Fig. 1).

### FST

**Fig. 2:**
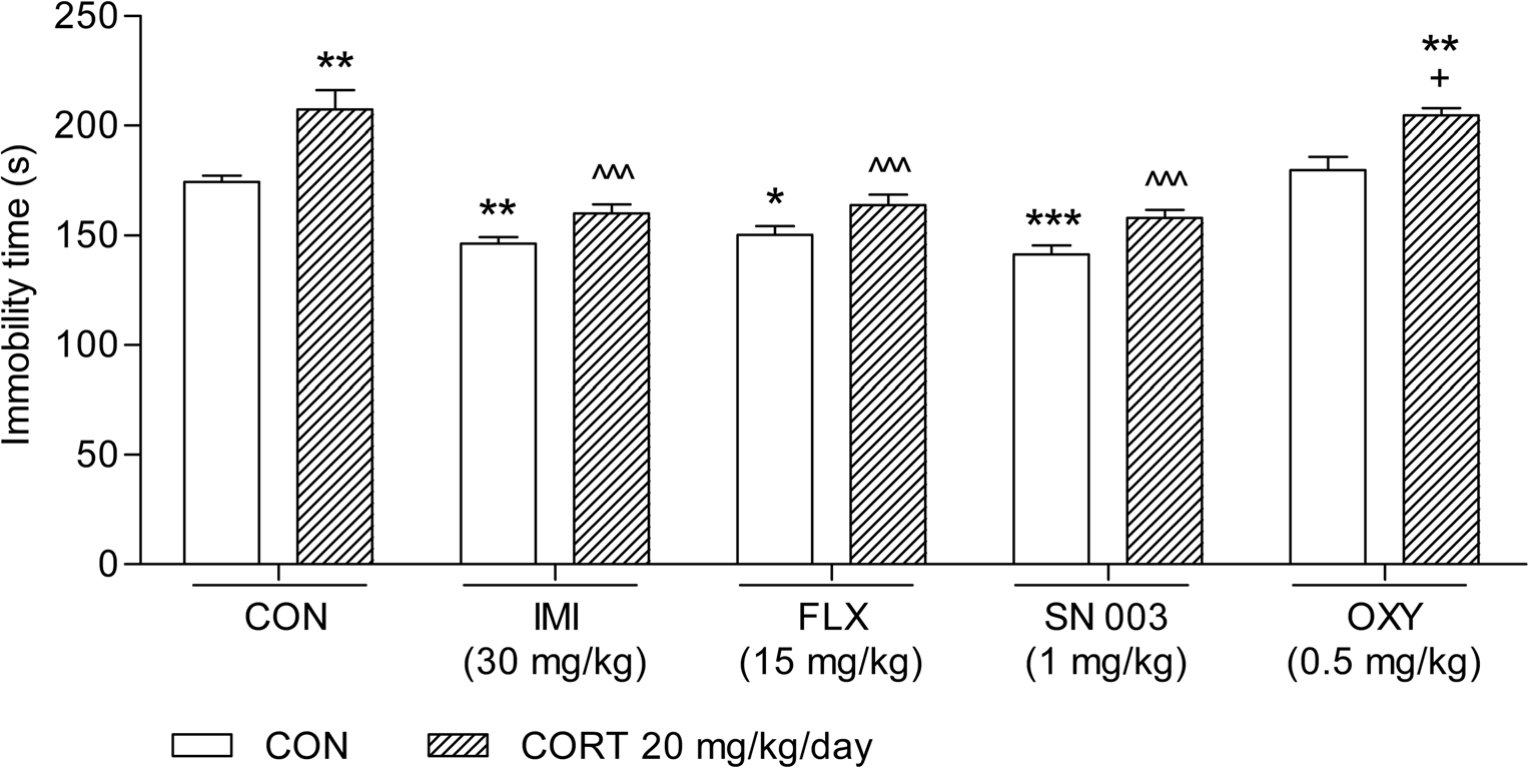
Influence of the administration of IMI (30 mg/kg), FLX (15 mg/kg), SN 003 (1 mg/kg), and OXY (0.5 mg/kg) on the behaviour of rats subjected to 14-day CORT (20 mg/kg/day) in the forced swim test (FST). The values represent the mean + SEM (*n* = 15 mice per group). ^^^*p* < 0.001 versus CORT, +*p* < 0.05 versus OXY, *p < 0.01, **p < 0.01, ***p < 0.001 versus CON (Tukey’s post hoc test)

As illustrated in Fig. 2, 14-day treatment with CORT at a dosage of 20 mg/kg/day considerably increased the immobility time of the animals tested (t(28) = 3.558;* p* = 0.0014). This effect was reversed by the administration of a single active (antidepressant) dose of IMI (30 mg/kg; F(3,56) = 24.42;* p* < 0.0001), FLX (15 mg/kg; F(3,56) = 18.81;* p* < 0.0001), and SN 003 (1 mg/kg; F(3,56) = 26.99;* p* < 0.0001). OXY at a dose of 0.5 mg/kg neither altered the immobility time of the rats nor abolished the pro-depressive effect elicited by CORT (F(3,56) = 8.444;* p* = 0.0001).

### CRF analysis

Serum CRF levels were 6.41 ± 0.28 pg/ml in the control group and 23.39 ± 1.13 pg/ml in the group that had received CORT at a dose of 20 mg/kg/day. A *t* test revealed the following statistics: t(28) = 14.49,* p* < 0.0001.

### Histopathology

**Fig. 3:**
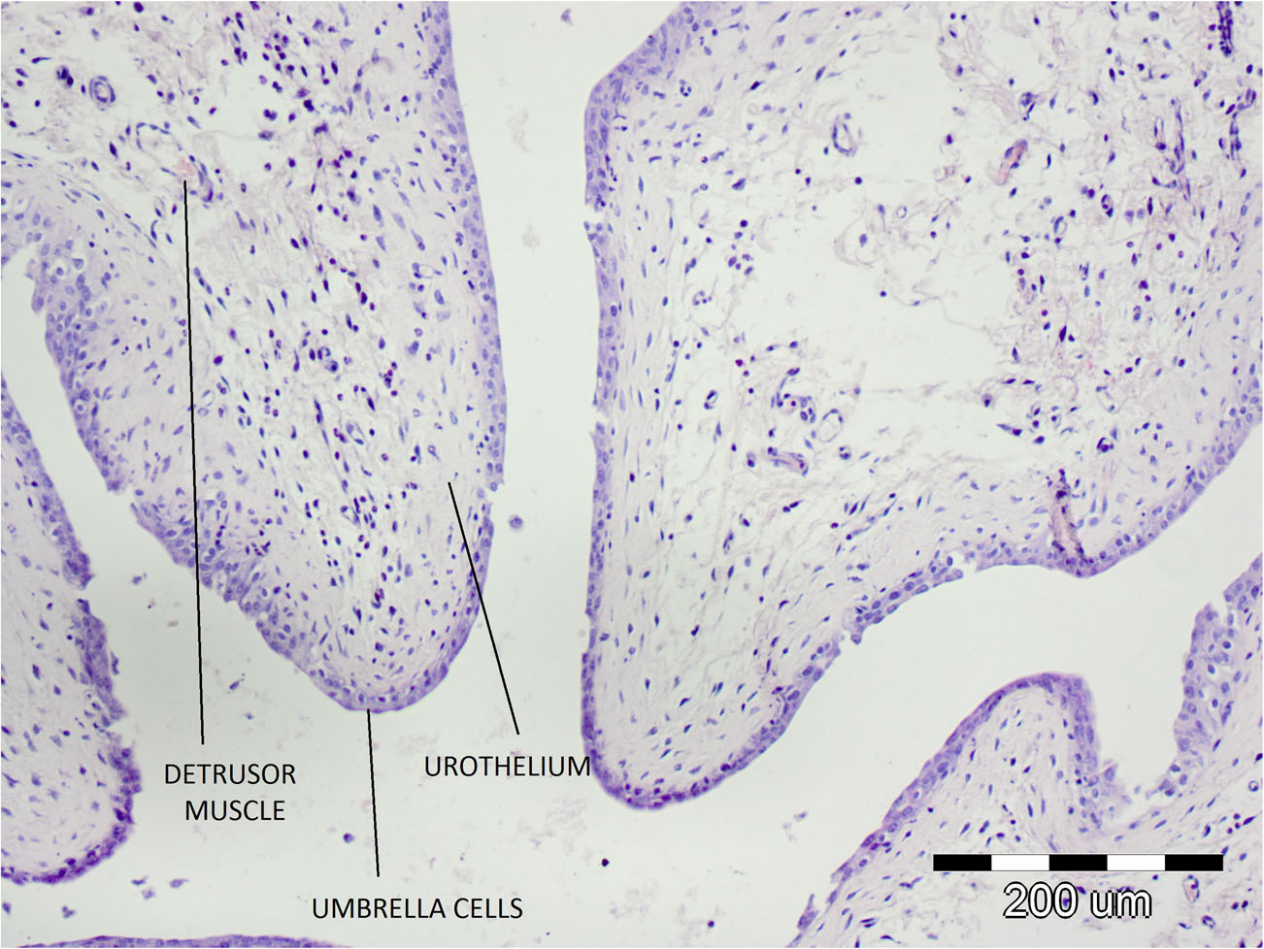
Microscopic examination of the saline-treated bladders

**Fig. 4:**
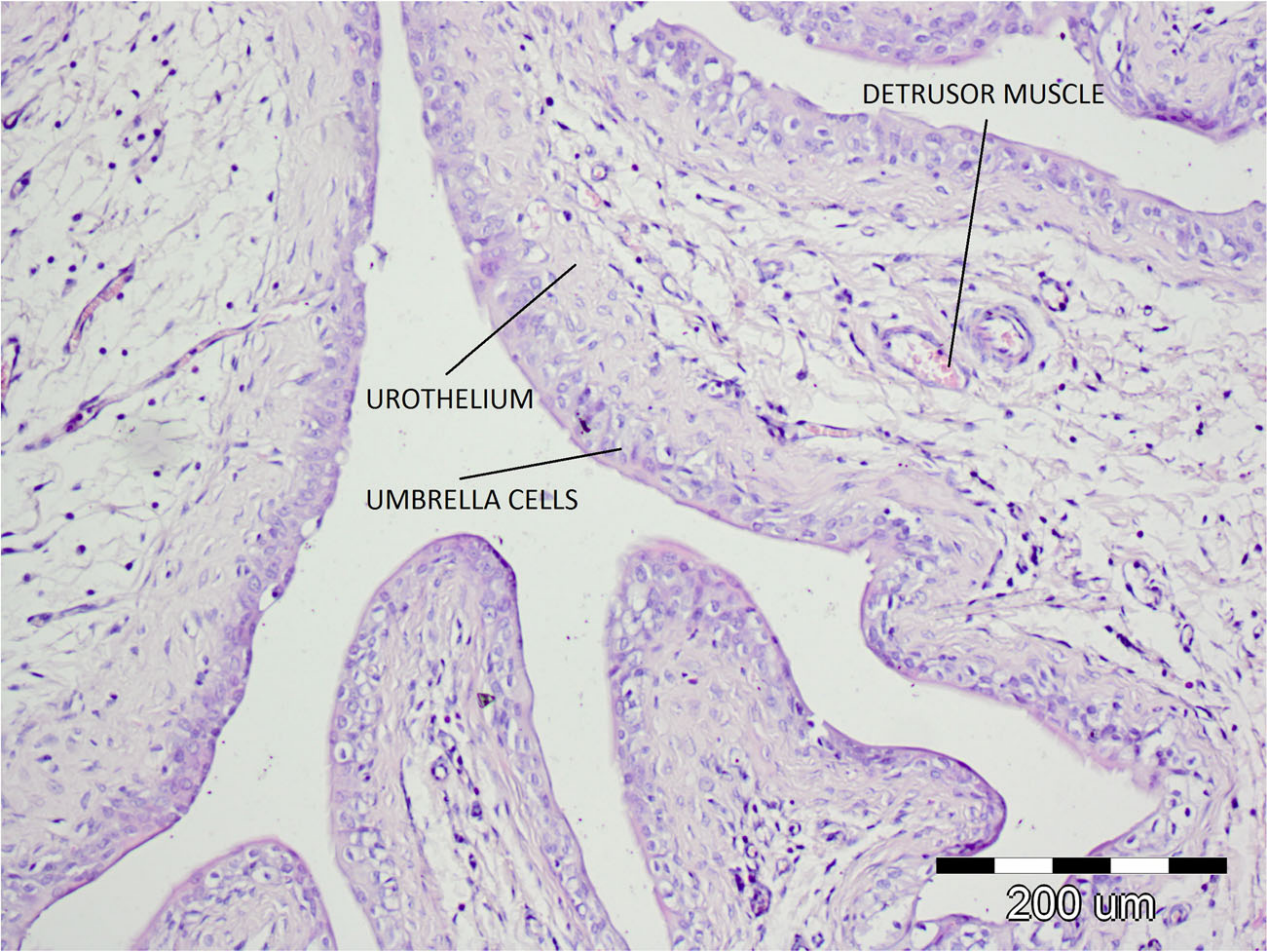
Microscopic examination of the corticosterone-treated bladders

In the histological specimens of the bladders from the tested animals subjected to CORT treatment, neither signs of bladder inflammation nor destructive changes in umbrella cells, urothelium, or detrusor muscle were observed (Figs. 3, 4).

## Discussion

The literature data provide evidence that the repeated administration of CORT via a parenteral route is a proper tool for obtaining an animal model of depression that corresponds, at least partially, to symptoms of clinical depression in humans [8]. This model is based on a generally recognised fact of the contribution of repeated stress and hypothalamic–pituitary–adrenal (HPA) axis hyperactivation to the development and manifestation of depressive disorders. Laboratory analyses revealed that plasma, urine, and cerebrospinal fluid cortisol concentrations are elevated in depressives [15]. Moreover, amongst people with Cushing’s disease (which is characterised by chronically high levels of cortisol), co-existing depression is unusually common [16]. Prolonged exposure to stress or corticosterone administration induces changes in the same brain areas (i.e. the hippocampus, amygdala, prefrontal cortex) as those that are afflicted in depressed patients [17].

As expected, 14-day administration of CORT injections at a dose of 20 mg/kg/day elicited a depressive phenotype on the FST, significantly reducing the mobility time of the animals tested. Similar results were published by other authors. This effect appears to be dose-dependent and affected by the duration of CORT treatment [8, 9]. Although the exact mechanism of the observed pro-depressive activity of CORT has not yet been explained, it is suspected that the exposure to exogenous CORT may modulate functioning of the serotonin system and the expression of 5-HT1A and 5-HT2A [7]. In addition, there are suggestions that the repeated administration of this steroid could induce morphological and/or neurochemical changes in the rat’s brain (as mentioned above) [17]. Despite the fact that CORT-treated animals usually gain much less weight than their control counterparts, this factor did not influence the results obtained in the Porsolt test [8, 9]. The results of our research were not affected by changes in the locomotor activity of rats either, as no significant differences in the overall locomotion between the groups tested were recorded in our study or in that by Gregus et al. [8]. According to the findings of Marks et al. [9], the depressive behaviour of rats subjected to the administration of CORT in the FST could not have been a result of weakened muscle strength. Moreover, in the case of the CORT animal model, it appears not to be important whether the Porsolt test was carried out as the one-day or the two-day version [9].

Significantly higher CRF levels observed in the CORT-treated group may be explained by dysregulation of the HPA axis induced by 14-day administration of CORT. It is well known that CRF is released in response to acute stress, and it promotes secretion of the adrenocorticotropic hormone (ACTH), which in turn increases the production of glucocorticoids in the adrenal cortex. Release of cortisol in humans and corticosterone in rodents acts via a well-described negative feedback loop and suppresses secretion of both CRF and ACTH. However, prolonged exposure to stress alters the functioning of the HPA axis and leads to the glucocorticoid-induced increase in CRF production. Dysregulation of the HPA axis with additional secretion of CRF and ACTH has been observed in numerous psychiatric-related disabilities, including depression [18].

In view of our present results and the outcomes of the recent study performed by Smith et al., the HPA axis plays an important role in the pathophysiology of OAB [19]. Furthermore, Smith et al. suggested that the subjective experience of OAB might also be determined by stress, and that stress reactivity in patients with OAB could be used as a factor allowing patient response to standard OAB therapy to be predicted. As our goal was focused on depressive behaviour only, we did not measure anxiety levels in the animals. Therefore, it cannot be excluded that the results obtained were also partially stress-affected. This fact notwithstanding, our findings are still valid and not markedly confounded, because a strong link between the pathophysiology of depression and anxiety has been demonstrated, and a number of patients suffer from mixed anxiety–depressive disorder [20].

We confirmed that SN 003, the CRF_1_ antagonist that displays >1,000-fold selectivity over CRF_2_ receptors, possesses antidepressant-like activity comparable with the typical antidepressant drugs (i.e. IMI and FLX). SN 003 suppresses CRF-induced ACTH release in vitro and is brain-penetrant. There are reports in the medical literature indicating that other agents belonging to CRF_1_ antagonists can significantly shorten the immobility time of animals in the FST as well [21]. However, it should be emphasised that the activity of different substances from this pharmacological group has been highly variable, depending on the experimental conditions and testing scheme. The number of days, depth and temperature of the water, the dose and route of administration could have been the main determinants for the expression of the antidepressant-like effect in the Porsolt test [21].

One of the aims of our work was to investigate if depression induced by a 14-day administration of CORT might lead to the development of DO symptoms. The results from cystometric studies confirm that the pro-depressive behaviour in animals, noted as a consequence of CORT treatment, may evoke the changes in cystometric parameters that are analogous to those observed in urodynamic diagnostics in humans, and they correspond to a diagnosis of DO. Consequently, an increase in BP, DOI, ANVC and FNVC, and a decrease in VV, ICI, BC, and VTNVC was recorded. At the same time, PVR value did not change significantly. As far as we know, this is the first study indicating that the depressive disorders may elevate DOI, an index considered to be the most reliable in the assessment of the severity of DO. It is broadly believed that DOI allows contraction activity of bladder detrusor to be assessed more precisely than the other parameters, such as ANVC, FNVC, MVP, ICI, BP, or BC [10].

Although administration of IMI, FLX and SN 003 managed to abolish the pro-depressive activity of the 14-day administration of CORT, only the inhibition of CRF_1_ by SN 003 reversed CORT-induced DO. This observation is in accordance with reports of other authors [22], who had proved that the selective antagonists of CRF_1_ reduce DO symptoms. An increase in VTNVC as a consequence of SN 003 administration is very important from a clinical point of view, as this parameter is generally considered the preclinical equivalent of the volume at the first involuntary detrusor contraction, and a reliable measure of treatment effectiveness for OAB. Elevation of VTNVC values leads to a reduced number of urinary incontinence episodes and decreased frequency of micturition [13]. SN 003, unlike OXY, did not have a significant influence on PVR. Such an observation suggests that SN 003 might improve urine storage, with no impairment of voiding function. A decrease in DOI, ANVC and FNVC induced by the CRF_1_ antagonist tested may indicate participation of CRF_1_ receptors in afferent mechanisms that regulate the micturition cycle. One of the most important findings of the present study is demonstration of the ability of OXY to reverse DO symptoms, as antimuscarinics are the established standard medical treatment for OAB.

According to the literature [23], chronic stress induces hyperactivity of the HPA axis and elevates CRF levels. Neurons that contain CRF receptors determine the HPA axis state and play a crucial role in the pathogenesis of depression. An increase in serum CRF levels in the animals exposed to CORT therapy that were tested in our research seems to be a common factor of the aetiopathogenesis of both depression and OAB [2], as it was shown that the brain areas (i.e. Barrington’s nucleus in the brainstem amygdala, the prefrontal cortex, the hippocampus) that respond to stress factors contain a high density of CRF_1_ receptors and take part in the regulation of the micturition reflex. Pre-clinical studies demonstrated that the CRF plays an important role in the conduction of afferent impulses from the urinary bladder. Skofitsch et al. [24] showed that the selective destruction of afferent C-fibres by capsaicin leads to a decrease in CRF expression. CRF may influence the micturition cycle via lowering the threshold for bladder afferent outflow. Consequently, it induces an increase in the contraction activity of the bladder detrusor. Moreover, CRF seems to be involved in the regulation of the micturition at the spinal cord level [25].

Finally, a histopathological analysis of the bladders of the tested rats subjected to CORT treatment did not reveal signs of bladder inflammation or destructive changes in umbrella cells, urothelium, or muscle layer.

Detrusor overactivity induced by CORT accurately reflects the OAB symptoms observed in humans, which is confirmed by the lack of histopathological lesions typical of interstitial cystitis. The animal models of induced hypersensitivity applied so far cannot be called OAB models because in their case DO is induced by an acute inflammatory reaction of the bladder, which does not constitute the basis for OAB pathophysiology. This can also be confirmed by the results of pathophysiological examinations of OAB patients, in whom no inflammatory lesions were found [26]. In the case of interstitial cystitis, cholinergic system function disorders were identified, together with changes in muscarinic receptors expression, which, in combination with inflammatory lesions and damage to the urothelium, may be the cause of the ineffectiveness of anticholinergics [27, 28]. Clinical studies have confirmed that a substantial percentage of interstitial cystitis patients are resistant to treatment with anticholinergics [29, 30]. However, the effectiveness of OXY in the reversal of CORT-induced DO found in the study is consistent with that observed in OAB patients.

In summary, our study provides new data on the association of depression with OAB. Three main findings should be particularly underlined:

1. Fourteen-day administration of CORT may induce DO symptoms in rats without producing any histopathological changes in the urinary bladder
2. CORT-induced DO responds to OXY
3. Inhibition of CRF_1_ receptors may reverse the symptoms of both depression and DO in animals exposed to CORT treatment

Thus, it seems that the CRF_1_ receptor could be an interesting target for OAB pharmacotherapy, particularly in patients with co-existing depression.
